# Learning from beautiful monsters: phylogenetic and morphogenetic implications of left-right asymmetry in ammonoid shells

**DOI:** 10.1186/s12862-019-1538-5

**Published:** 2019-11-13

**Authors:** Romain Jattiot, Emmanuel Fara, Arnaud Brayard, Séverine Urdy, Nicolas Goudemand

**Affiliations:** 1 Biogéosciences, UMR 6282, CNRS, Université Bourgogne Franche-Comté, 6 boulevard Gabriel, 21000 Dijon, France; 2 Univ. Lyon, ENS de Lyon, CNRS, Université Claude Bernard Lyon 1, Institut de Génomique Fonctionnelle de Lyon, UMR 5242, 46 allée d’Italie, F-69364 Lyon Cedex 07, France

**Keywords:** Ammonoid shell, Left-right asymmetry, Pathology, Development, Phylogeny, Morphogenesis

## Abstract

**Background:**

Many pathologies that modify the shell geometry and ornamentation of ammonoids are known from the fossil record. Since they may reflect the developmental response of the organism to a perturbation (usually a sublethal injury), their study is essential for exploring the developmental mechanisms of these extinct animals. Ammonoid pathologies are also useful to assess the value of some morphological characters used in taxonomy, as well as to improve phylogenetic reconstructions and evolutionary scenarios.

**Results:**

We report on the discovery of an enigmatic pathological middle Toarcian (Lower Jurassic) ammonoid specimen from southern France, characterized by a pronounced left-right asymmetry in both ornamentation and suture lines. For each side independently, the taxonomic interpretations of ornamentation and suture lines are congruent, suggesting a *Hildoceras semipolitum* species assignment for the left side and a *Brodieia primaria* species assignment for the right side. The former exhibits a lateral groove whereas the second displays sinuous ribs. This specimen, together with the few analogous cases reported in the literature, lead us to erect a new forma-type pathology herein called “forma janusa” for specimens displaying a left-right asymmetry in the absence of any clear evidence of injury or parasitism, whereby the two sides match with the regular morphology of two distinct, known species.

**Conclusions:**

Since “forma janusa” specimens reflect the underlying developmental plasticity of the ammonoid taxa, we hypothesize that such specimens may also indicate unsuspected phylogenetic closeness between the two displayed taxa and may even reveal a direct ancestor-descendant relationship. This hypothesis is not, as yet, contradicted by the stratigraphical data at hand: in all studied cases the two distinct taxa correspond to contemporaneous or sub-contemporaneous taxa. More generally, the newly described specimen suggests that a hitherto unidentified developmental link may exist between sinuous ribs and lateral grooves. Overall, we recommend an integrative approach for revisiting aberrant individuals that illustrate the intricate links among shell morphogenesis, developmental plasticity and phylogeny.

## Background

Due to their abundance, high evolutionary rates and widespread distribution, ammonoids represent an iconic fossil group of mollusks. Their study contributes to our understanding of mollusk evolution and biology. For example, much can be learned from the study of ammonoids when addressing the intricate links between morphogenesis, developmental plasticity and phylogeny in mollusks. In this regard, many pathologies that modify the shell geometry and ornamentation of ammonoids are known from the fossil record. These pathologies can shed light on ammonoid paleobiology and paleoecology, for instance by providing information on their potential predators and predation modes [1–8], on the impact of parasites on shell morphology [2–4, 9–11], or on putative functions of ornamentation (e.g., as antipredatory trait [12]). Since they occur in many different forms, ammonoid shell pathologies are classified into categories called forma-types; most of which are described in two major review works [4, 13].

In addition, pathologies in ornamental patterns constitute “natural experiments” that may reveal crucial information about the developmental mechanisms underlying healing processes, as well as those taking place during regular shell secretion [4, 14–16]. For instance, on a ventrally-keeled ammonoid shell, if the keel is lost due to an injury, the ribs that are usually restricted to the flanks may cross the venter and replace the keel on the damaged shell: a phenomenon named as ornamental compensation [14, 15]. Similarly, on a specimen of *Paraceratites* ([17], fig. 4c, d) that displays a mid-venter injury, the keel is lost on the damaged shell, and the usually small ventrolateral tubercles (observed on the pre-damaged part of the shell) are replaced by massive spines. Such an enhancement of the ornamentation is in agreement with the removal of an inhibitory zone bordering the ventral keel as predicted by generalized reaction-diffusion models [18]. This is also compatible with a change in the mantle elastic properties of the injured venter, assuming that a scar leads to an increase in bending stiffness of the mantle at this location. Indeed, a mechanical model [19] suggests that mollusk spines are likely to grow in regions of relative low bending stiffness along the aperture and that the larger the gradient of local bending stiffness along the aperture, the sharper the resulting spines. Hence, the local, injury-induced increase of bending stiffness on the ammonoid venter would have exacerbated the gradient of bending stiffness along the aperture and resulted in the growth of massive spines (instead of tubercles) in the low-bending-stiffness ventrolateral region on both sides of the scar.

Morphogenetic studies, in particular theoretical studies, can help distinguish pathologies from regular intraspecific variation. Without developmental considerations, many pathological specimens presenting such ornamental compensation, termed “forma circumdata” [20], could be mistaken for regular taxa, or misinterpreted as atavistic individuals [16, 21, 22]. One of the most striking examples of such misinterpretations is the invalid ammonoid subfamily “Monestieriinae” [22], which is based on a pathological Grammoceratinae [16]. Therefore, the study of ammonoid pathologies is essential not only for exploring the developmental mechanisms of molluscan shells but also for assessing the value of some morphological characters used in taxonomy.

Here, we describe an enigmatic middle Toarcian (Early Jurassic) ammonoid specimen that shows a rare pathology characterized by a marked left-right asymmetry in ornamentation along the entire shell. Although ammonoid pathologies described in terms of ornamental compensation are relatively frequent, pathologies affecting the ornamentation in the absence of clear evidence of injury or parasitism are indeed particularly rare. We compare this specimen to the few other ammonoid specimens described in the literature that exhibit a similar pathology. We then discuss the implications of such specimens for ammonoid taxonomy, phylogeny and morphogenesis of ornamentation, in particular the possible developmental link between sinuous ribs and lateral grooves.

## Geological setting

**Fig. 1:**
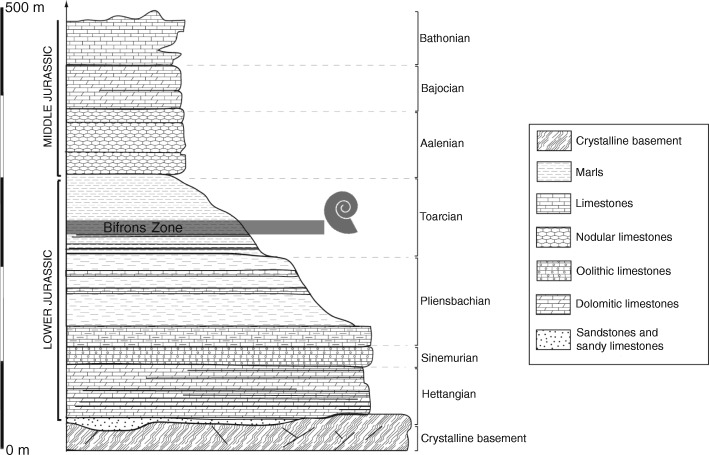
Synthetic stratigraphy of Cénaret locality with the position of the Bifrons Zone, the zone where the asymmetric ammonoid from Cénaret is assumed to come from (modified from [23])

The locality of Cénaret is located near the township of Barjac (Lozère Département), in the northern part of the Causses Basin, on the southern border of the Massif Central. The Mesozoic sedimentary deposits filling the Causses Basin lie in unconformity with the ante-Hercynian and Hercynian crystalline basement (Fig. 1). The filling of the Causses Basin is due to the progressive advancement of the Tethys Sea, ca. 200 million years ago.

In the vicinity of Cénaret, the sedimentological succession mainly consists of Domerian (upper Pliensbachian) to Bathonian deposits (Fig. 1). The lower Toarcian is composed of black shales in which ammonoids (e.g., *Harpoceras serpentinum*) and fish remains (e.g., *Leptolepis coryphaenoides*) are abundant. Middle and upper Toarcian sediments consist of blue marls containing a highly diversified marine fauna. The benthic fauna is represented by common gastropods (e.g., *Turbo*), bivalves (*Ledarostralis*, *Pecten pumilus*) and crinoids. The pelagic fauna includes very abundant ammonoids [24], nautiloids and rare ichthyosaur remains. These marls are overlain by Aalenian, Bajocian and Bathonian limestones (Fig. 1).

The studied specimen was found as float in the middle Toarcian marls and most likely belongs to the uppermost part of the Bifrons Zone (Fig. 1) based on the geomorphology of the outcrop [24].

## Results

### Description of the specimen

#### Shape and ornamentation

**Fig. 2:**
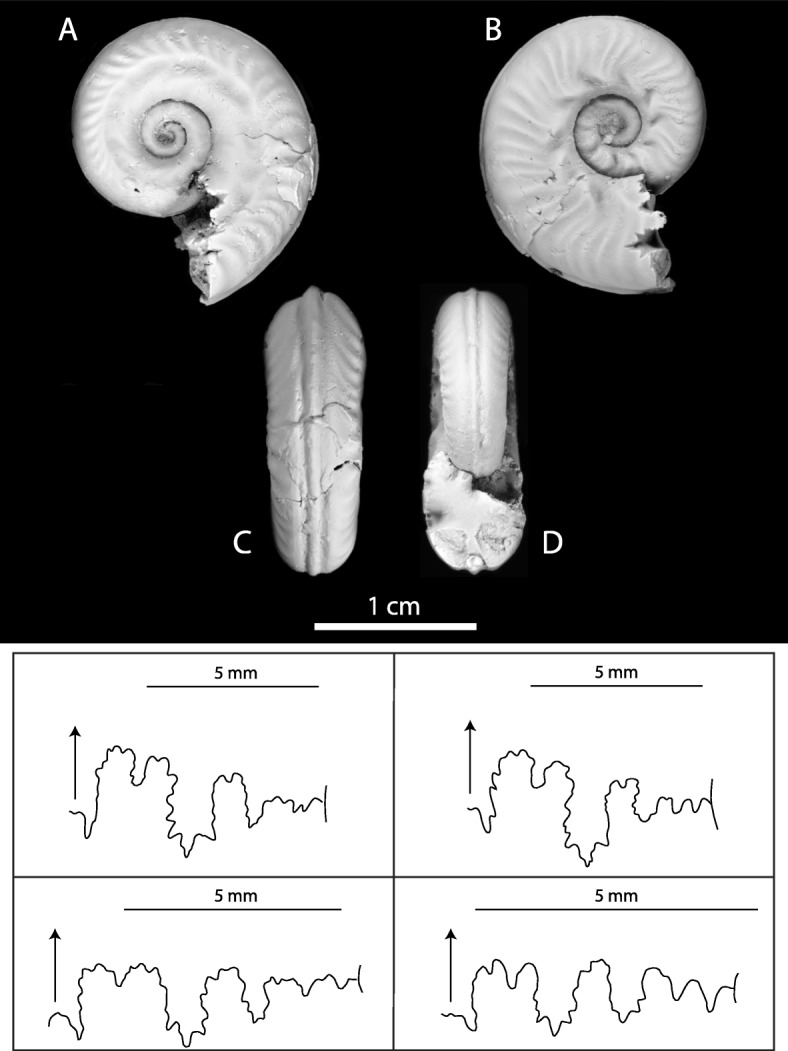
Asymmetric specimen from Cénaret (UBGD 28012). **a** Left side, assigned to *Hildoceras semipolitum*. **b** Right side, assigned to *Brodieia primaria*. **c** Ventral view and **d** apertural view. **e** Suture line of the left side and **f** suture line of the right side. **g** Suture line of a *Hildoceras semipolitum* specimen [25] and **h** suture line of a *Haugia variabilis* specimen [25]

The shell is moderately evolute and slightly compressed. The umbilical shoulders are rounded and the umbilical wall is weakly inclined. The whorl section is quadrangular and the ventral part is clearly tricarinate, with a central keel in between two well-defined grooves forming two secondary keels (Fig. 2c, d). The body chamber is not preserved. Given the number of whorls preserved (i.e., about three), the specimen is considered to be a juvenile to sub-adult, and not a hatchling.

On the left side (Fig. 2a), a well-defined longitudinal groove is visible on the middle of the flank all along the coiling. The ribs are moderately strong, rursiradiate, concave and distant. The generic determination is not questionable since the presence of a longitudinal groove on the middle of the flank is characteristic of the genus *Hildoceras*. The umbilical wall is clearly less inclined and more rounded than in *Hildoceras apertum* [26]. Both *H*. *bifrons* [27] and *H*. *semipolitum* [28] are characterized by a well-defined longitudinal groove located at mid-distance between the successive umbilical sutures [29]. Although ammonoid workers diverge on their interpretations of the distinction between *H. bifrons* and *H. semipolitum* [29], the lack of perceptible ribbing on the inner whorls strongly suggests that this side corresponds to the species *H. semipolitum*.

On the right side (Fig. 2b), the ribs are weakly flexuous, rursiradiate and acute on the ventral edge. They are bifurcated in the inner whorls and at the beginning of the last preserved whorl; then trifurcated. These polyfurcated ribs start with a slender and prorsiradiate umbilical tubercle and they alternate with rare simple ribs. We identified this side as typical of the species *Brodieia primaria* [30], based on the rursiradiate, polyfurcated ribs combined with weak and prorsiradiate umbilical tubercles.

#### Suture lines

On both sides of the specimen, the suture lines are moderately complex with short, stocky lobes and saddles. The suture lines of the two sides are relatively similar but there is a significant difference near the umbilical edge (Fig. 2e, f). Pathologies generally do not modify the suture lines [4, 13], as even a conspicuous change in ornamentation such as the one induced by a “forma circumdata” (ornamental compensation) may not affect the suture lines [15]. A few pathologies may affect the suture lines, the most pronounced being the “forma juxtabolata” pathology [20], characterized by the displacement to one side of the siphuncle and ventral lobe of the suture.

On the left side of the specimen, the suture lines (Fig. 2e) are similar to that of *Hildoceras semipolitum* (Fig. 2g; [25]). Suture lines of *Hildoceras bifrons* [31] markedly differ in displaying among other features, a larger lateral lobe. Hence, the suture lines corroborate our interpretation of the ornamentation of the left side as corresponding to *H*. *semipolitum*.

On the right side of the specimen, the suture lines (Fig. 2f), especially near the umbilical suture, are similar to the few illustrated suture lines of *Haugia variabilis*, especially that of a small-diameter specimen (Fig. 2h; [25]). *Brodieia* is commonly considered as the microconch of *Haugia* [29, 31, 32], and therefore both genera have nearly identical suture lines at small, comparable size.

Thus, this pathological specimen from Cénaret is characterized by a pronounced left-right asymmetry in both ornamentation and suture lines. The ontogeny of the specimen indicates that the asymmetry was not immediately fatal to the embryo or hatchling; therefore, the shell was functional during growth, despite the significant asymmetry of its shell.

For each side considered independently, the taxonomic interpretations of ornamentation and suture lines are congruent, suggesting a *Hildoceras semipolitum* species assignment for the left side and a *Brodieia primaria* species assignment for the right side. Note that our taxonomic identifications of the specimen were confirmed by two ammonoid workers (I. Rouget and P. Neige) who identified the two sides separately in a blindfold test.

### Systematic paleontology

Ammonoidea Zittel 1884

Ammonitina Hyatt 1889

Hildocerataceae Hyatt 1867

Hildoceratidae Hyatt 1867

#### Left side

Hildoceratinae Hyatt 1867

*Hildoceras* Hyatt 1867

*Hildoceras semipolitum* Buckman 1902

#### Right side

Phymatoceratinae Hyatt 1867

*Brodieia* Buckman 1898

*Brodieia primaria* Schirardin 1914

### Comparison with other pathological specimens

Ammonoid pathologies are classified into categories called forma-types ([20], see [4, 13] for reviews). The majority of the forma-types previously described in the literature are thought to have been induced by either injury or parasitism. In Hengsbach’s classification [13], only the “forma syncosta” pathology could not be linked to an exogenic cause and hence was exclusively associated with a putative genetic mutation. The pathological specimen from Cénaret does not correspond to any forma-type pathology described to date [2–4, 13]. To our knowledge, no one ever defined a forma-type for ammonoids showing a marked left-right asymmetry in morphology that matches two distinct species, although comparable but rare specimens have been previously illustrated [29, 33, 34]. We discuss these specimens below.

#### Specimen from Tintant [33]

**Fig. 3:**
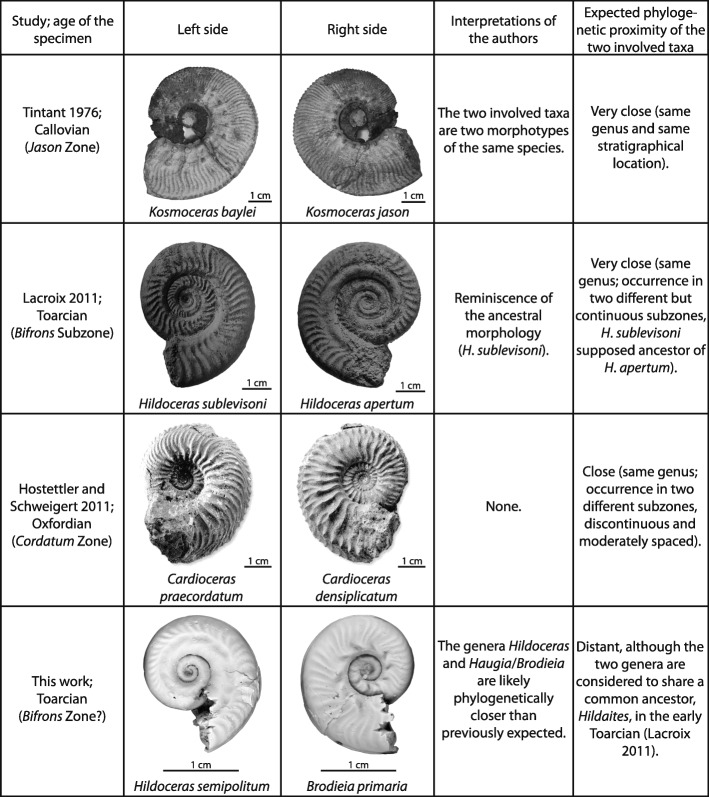
Description of “forma janusa” specimens

Tintant [33] identified the left side of this specimen as typical of *Kosmoceras baylei* [35], which displays two rows of tubercles (one umbilical and one lateral; Fig. 3), and he assigned the right side to *Kosmoceras jason* [36], which shows only one row of tubercles (umbilical position; Fig. 3). Our analysis of the specimen (UBGD 277447) confirms Tintant’s [33] identifications. The two species involved, *K. baylei* and *K. jason*, are known to be contemporaneous in the Callovian Jason Zone. This contemporaneity led Tintant [33] to suggest that these two species may represent two morphotypes of a single species.

#### Specimen from Lacroix [29]

The right and left sides of this specimen (Fig. 3), found in the Apertum Horizon (Bifrons Subzone), correspond to the taxa *Hildoceras apertum* and *Hildoceras sublevisoni* [29], respectively. These two species are not known to be contemporaneous, *H*. *sublevisoni* occurring in the Sublevisoni Subzone [27] and *H*. *apertum* usually occurring in the subsequent Bifrons Subzone. Ammonoid workers agree that *H*. *sublevisoni* is probably the ancestor of *H*. *apertum* [26, 29, 37]. The occurrence of these two morphologies on the same specimen may be related to this close phylogenetic relationship.

#### Specimen from Hostettler and Schweigert [34]

According to Hostettler and Schweigert [34], the left and right sides of this specimen match the definition of *Cardioceras* (*Pavloviceras*) *praecordatum* and *Cardioceras* (*Vertebriceras*) *densiplicatum,* respectively (Fig. 3). These taxa are not contemporaneous, as they are usually found in two discontinuous subzones: the Praecordatum Subzone (earliest Oxfordian) and the Vertebrale Subzone (earliest middle Oxfordian). Interestingly, the studied specimen was found in the Cordatum Zone [34] in between these two subzones. Although the authors did not discuss the potential phylogenetic implications of this specimen, we stress the close phylogenetic relationship between the two species, similarly to Tintant and Lacroix’s specimens.

#### Specimen from this study

**Fig. 4:**
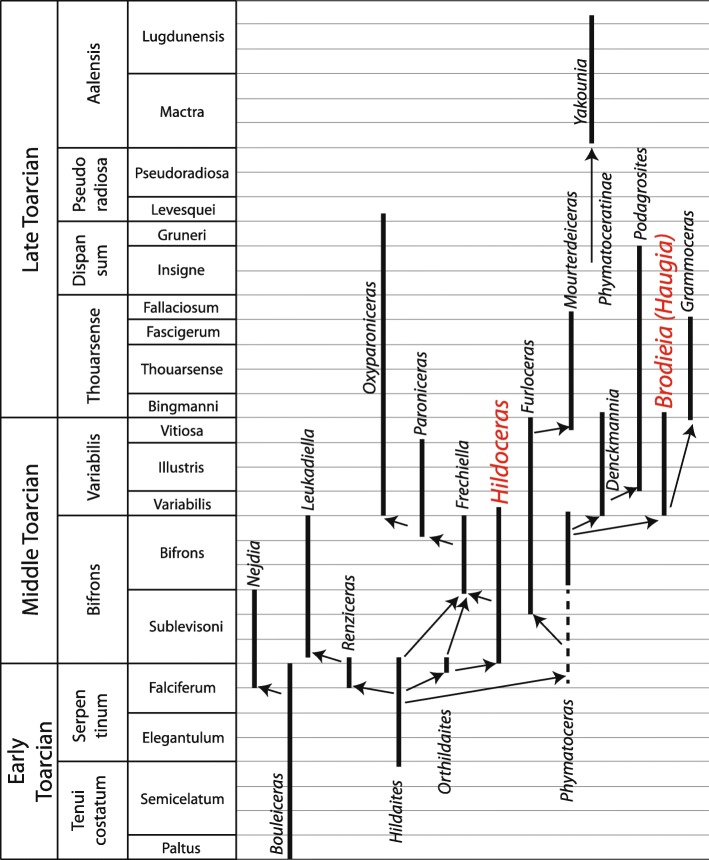
Phylogenetic hypothesis for some Toarcian clades, including *Hildoceras* and *Brodieia* / *Haugia* (in red; modified from [29])

For the Cénaret specimen, the left side corresponds to *Hildoceras semipolitum* and the right side to *Brodieia primaria*. The phylogeny of northwest European Toarcian ammonoids is still debated but the classical phylogeny of Lacroix ([29]; Fig. 4) hypothesizes a rather large phylogenetic distance between *Hildoceras* and *Haugia*/*Brodieia* (*Brodieia* being the microconch of *Haugia* [29, 31, 32]). According to this hypothesis, the early Toarcian genus *Hildaites* is the common ancestor of the early middle Toarcian *Hildoceras* and the late middle Toarcian *Haugia*/*Brodieia*, via *Orthildaites* and *Phymatoceras*, respectively [29]. This hypothesis implies a ghost lineage of *Phymatoceras*, as this genus is known neither from the latest early Toarcian nor the early middle Toarcian (Fig. 4). The species assigned on the two sides of the specimen from Cénaret (Fig. 2) are either not regarded as close relatives in the empirical phylogeny proposed by Lacroix [29], or are not included in the recent cladistic hypothesis of Bardin et al. [38]. This pathological specimen suggests however that the genera *Hildoceras* and *Haugia/Brodieia* may be phylogenetically closer than previously expected. Interestingly, in Lacroix’s phylogenetic scheme [29], *Hildoceras* and *Haugia/Brodieia* appear to coexist during a very short time interval (the lowermost part of the Variabilis Zone; Fig. 4), a contemporaneousness that is well supported by other works [39, 40]. The congruence between ornamentation and suture line, as well as the occurrence in contemporaneous zones, suggest a potential ancestor-descendant relationship in which the older *Hildoceras* would be the ancestor of the younger *Haugia*/*Brodieia.*

### Definition of the new forma-type pathology “forma janusa” and phylogenetic implications

Given the peculiar shell morphology mentioned above, we erect a new forma-type pathology here named “forma janusa” (from Janus, the two-faced Roman god) for specimens that display a left-right asymmetry of the entire shell in the absence of clear evidence of injury or parasitism, each side corresponding to the diagnoses of two distinct species. We include the aforementioned specimens [29, 33, 34] and the individual from Cénaret in the “forma janusa” pathology (Fig. 3). None of these specimens presents any clear evidence for injury or parasitism. The Cénaret specimen shows an asymmetry that is visible in the earliest ontogenetic stages, as confirmed by Scanning Electron Microscope (SEM) observations on the inner whorls and the protoconch of both sides (see Additional files 1, 2).

**Fig. 5:**
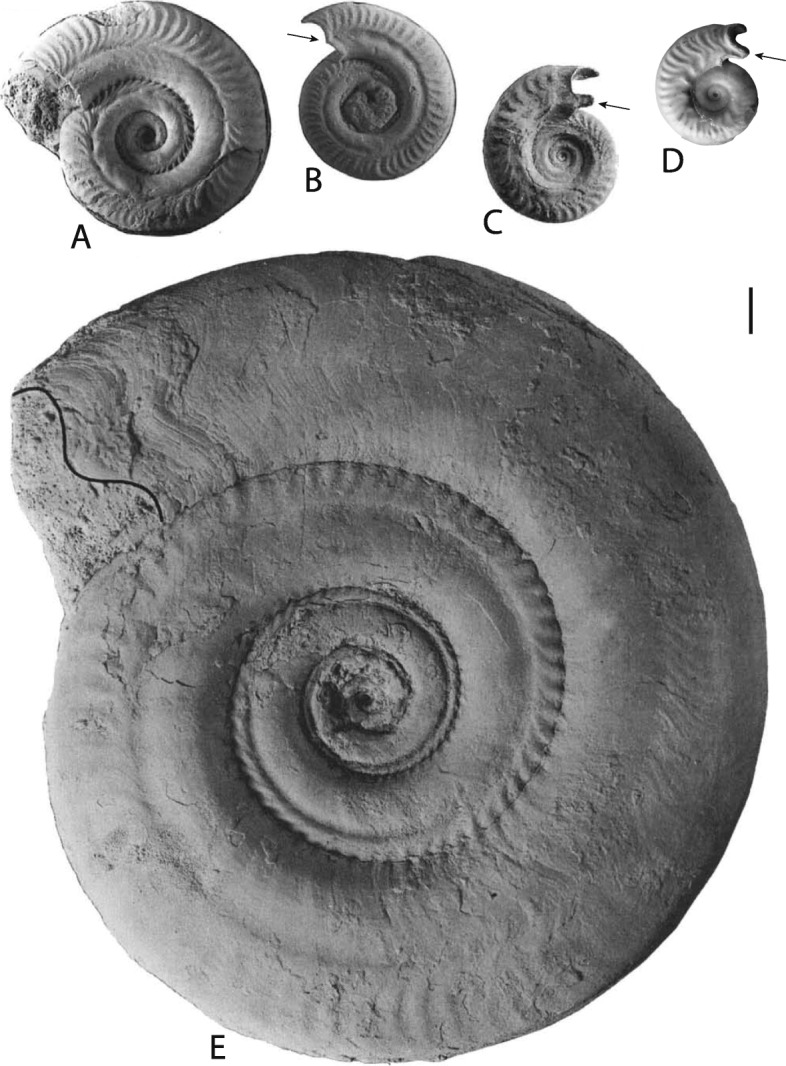
Growth lines and shell lappet morphology in *Hildoceras* and *Brodieia*. **a** *Hildoceras bifrons*, FSL 299509, [88]. **b** *Hildoceras bifrons*, FSL 169352, [88]. **c** *Hildoceras lusitanicum*, FSL 525637[39]. **d** *Brodieia courryense*, AIRV 8600 (coll. Boursicot). **e** *Hildoceras bifrons*, FSL 11890, [88]. Arrows indicate the lappets. Scale bar is 10 mm

Conversely, the “forma janusa” morphology does not include cases where one side only can be assigned to a known species and the other side displays irregularities or an association of traits revealing an injury or parasitism and only superficially resembling another taxa (e.g., [4], fig. 157; [41], fig. 5i-j).

Regardless of whether “forma janusa” specimens are due to an exogenic or endogenic cause, such specimens are potentially informative for phylogenetic reconstructions. Indeed, an accurate matching of the ornamentation (and possibly suture lines) on the altered side with the ornamental scheme (and possibly suture lines) of an actual species does not seem coincidental, especially given that these specimens are found in biostratigraphical zones that are contemporaneous or sub-contemporaneous to the zones where the species corresponding to the two diagnosed sides are found. Overall, the few known examples of “forma janusa” specimens suggest that the two displayed species document a segment of a same evolutionary lineage.

## Discussion

### Left-right asymmetry, expression of a developmental plasticity

Most bilaterians exhibit by definition a left-right bilateral symmetry, or approximately so. Since growth depends on a complex interplay between environmental, developmental and genetic processes, some degree of asymmetry is expected [42]. In this regard, a large body of literature deals with the common patterns of small, random deviations from the perfect bilateral symmetry known as fluctuating asymmetry [43–46]. Yet, the kind of asymmetry observed in “forma janusa*”* specimens corresponds to very rare events whereby the amplitude of the asymmetries and their nature would otherwise justify classification in two different taxa. Among bilaterians, there are rare occurrences of other spectacular asymmetries, for instance in bilateral gynandromorphic individuals (e.g., [47]), but to our knowledge nothing similar to “forma janusa*”* has ever been reported in mollusks. On the other hand, local shell asymmetries are common in fossil cephalopods. Their usual interpretation is that they were induced by injuries (e.g., [1–16, 48]). Specimens like that of Cénaret that exhibit a complete left-right asymmetry without any visual trace of post-larval injury are very rare. Likewise, numerous studies have described ammonoids with asymmetrical suture lines (e.g., 20, 49–58], but none of them for which both suture lines matched those of two different taxa, as is the case of the Cénaret specimen. Furthermore, it is not known whether these previously described asymmetrical suture lines were present throughout ontogeny, nor whether they were associated with asymmetrical ornamentation. Hence, the Cénaret specimen seems to stand out as an extreme case of left-right asymmetry that was presumably not induced by a post-larval injury.

Shell ornamentation and suture lines are two of the main groups of diagnostic characters used in ammonoid taxonomy. These sets of characters are usually considered as being independent. To date, our understanding of shell and suture development is very limited, but the integrated changes observed both in the suture lines and shell ornamentation in the Cénaret specimen point out that these two characters may not be as independent as previously assumed. In extant gastropods, proteomic and transcriptomic studies revealed an unsuspected molecular modularity and diversity of the shell-forming mantle tissue and suggested that the high degree of spatial modularity among distinct sets of genes may explain the high evolvability of the molluscan shell over evolutionary timescales [59, 60]. The “forma janusa” specimens described here also display a strong developmental plasticity and a high level of integration, with potential implications for our understanding of ammonoid phylogenetic relationships. This supports the hypothesis that a marked developmental plasticity may have allowed ammonoid clades to radiate rapidly and profusely as environmental changes opened up new ecological niches where new variants were sorted and diverged [61, 62]. Finally, some aspects of shell ornamentation appear as potentially plastic features in ammonoids. Thus, this calls for caution when defining ammonoid taxa and putative phylogenetic relationships based on a single set of ornamental features. A thorough assessment of the ornamental intraspecific variability may help to circumvent this problem.

More generally, the wide morphological diversity observed in modern mollusks has long been thought to be explained by differences in Hox gene interactions and expressions or changes in their downstream genes (e.g., [63]). Yet, all available evidence so far suggests that Hox genes are not expressed in the larval mantle of recent cephalopods [64, 65], contrarily to all other clades of mollusks. Clearly, these important questions require more investigation.

### Correlation of shell characters and evo-devo of the molluscan shell

Most of what is currently known about the development of the ammonoid shell ornamentation derives from empirical studies that have highlighted the non-independence of some shell characters, as well as from theoretical studies emphasizing the developmental constraints that may be at work in the evolution of the molluscan shell. For instance, the amplitude of ribs has been shown to covary with the aperture shape and degree of whorl overlap within species, a general trend coined as Buckman's first law of covariation by Westermann [66, 67]. Usually, faint ribs are associated with compressed apertures and involute coiling, while strong ribs are associated with depressed apertures and evolute coiling. These patterns of intraspecific variation have been observed in Triassic [68–77], in Jurassic [66] and in Cretaceous [78] ammonoids. As these patterns of covariation have been observed in phylogenetically distant ammonoids at several different time periods, they represent evolutionary convergences that stem from the developmental constraints imposed by accretionary growth [19, 79–83].

These covariations have been recently explained using a mechanical model, whereby ammonoid shells act as biomechanical oscillators [82, 83]. More generally, two types of dynamic models have been proposed to account for the morphogenesis of ornamentation in ammonoids: lateral inhibition (chemical) models and mechanical models (see review in [80]). These two types of models have in common a feedback mechanism that allows for the simulation of oscillations: temporal oscillations akin to commarginal ribs, spatial oscillations akin to anti-marginal ribs or keels, or combined temporal and spatial oscillations akin to spines or tubercles.

### Morphogenesis of lateral ribs and spines

The occurrence of “forma janusa” specimens, as described above, suggests that “types” of ornamental features that are usually considered as very different may actually correspond to variations on a same developmental process. Rudraraju et al. [79] recently developed a mechanical model that combines shell secretion and mantle morpho-elasticity and showed that the location, number and amplitude of longitudinal ribs and spines depend on three parameters: 1. the length of the actively-secreting mantle in the growth direction; 2. the kinetics of volumetric growth of the mantle that results in an overall increase in mantle edge perimeter if mantle thickness is kept constant and; 3. the local curvature of the previously secreted shell that imposes rigid constraints on how the soft growing mantle tissue can deform at each growth step. In extant bivalves, longitudinal ribs have been linked to the presence of raised and thick portions of the mantle surface called *corposa spinosa* [84, 85]. In the gastropod *Nucella ostrina*, the mantle margin has been observed to be scalloped or ruffled but not particularly thickened [86]. Each longitudinal rib in *Nucella ostrina* has been shown to be associated with a tongue-shaped extension of the mantle in the growth direction [86]. Longitudinal ribs in this dimorphic gastropod were produced by changes in size of the active mantle and epithelial cell morphology [86], the ribbed shells showing a larger elongation in the direction of growth of the outer epithelium as well as an increase in cell height compared to smooth shells of the same species. Similarly, intraspecific variation in spine length in another neogastropod, *Hexaplex trunculus,* seems to be dependent on the variation in length of the outer mantle fold in the growth direction along the mantle edge [80]. Additionally, the ruffled mantle edge of the gastropod *Haliotis asinina* is also clearly visible and each undulation is spatially associated with a longitudinal rib, although not commented upon by the authors ([59], fig. 4; [87], fig. 3). Therefore, the presence, amplitude and number of longitudinal ribs and spines along the aperture of several bivalve and gastropod species studied so far may be linked to the degree of mantle edge scalloping (and potentially further differentiation of epithelial cells). Although Rudraraju et al. [79] did not investigate the theoretical consequences of varying the active mantle length along the mantle edge, they show that the active mantle length is negatively correlated with the number and amplitude of emerging crests and valleys, all else being equal ([79], figs. 7, 8). Mantle undulations also seem associated with actin filaments in *Haliotis asinina*, which are mostly oriented perpendicularly to the mantle edge ([87], fig. 4). It is interesting to note that actin filaments could potentially create spatial variation in local relative bending stiffness along the mantle edge. Similarly, it is expected that spatial variation in mantle thickness, as observed in *corposa spinosa* in bivalves, would result in spatial variation in local relative bending stiffness along the mantle edge. From a theoretical point of view, spines and other longitudinal ribs are indeed more likely to grow in regions of low relative bending stiffness [19]. In our opinion, if the mantle margin exhibits spatial variation in its length (scalloped margin) and/or spatial variation in its thickness (*corposa spinosa*), two theoretical parameters could control the morphogenesis of longitudinal crests and valleys: 1. the variation in the length of the active mantle margin [79]; 2. the spatial variation in local bending stiffness of the mantle [19]. New experiments are needed to decipher in each case which theoretical parameter would account for the morphogenesis of longitudinal ornamentation and its intraspecific and interspecific variation in gastropods, bivalves and ammonites.

### Morphogenesis of longitudinal grooves

To our knowledge, there is currently no model accounting specifically for the formation of longitudinal grooves on the flank. Yet, similarly to longitudinal ribs, longitudinal grooves could correspond to local variation of the active length of the mantle in the growth direction or to changes in the relative bending stiffness along the mantle edge.

There is some evidence that ammonoids with sinuous ribs and/or longitudinal grooves also exhibited sinuous growth lines (Fig. 5). This indicates not only that sinuous ribs were commarginal (parallel to the aperture) but also that sinuous ribs and longitudinal grooves could be systematically associated with out-of-plane apertures, with a lappet-like extension of the mantle (Fig. 5). It is thus tempting to hypothesize that variation in the length of this mantle lappet in the growth direction would account for the formation of a longitudinal groove, the longer (or the stiffer) the mantle lappet, the deeper the groove.

Moreover, other aspects of the shell curvature (in particular the curvature of the umbilical wall and the distance of the lappet-like extension from the umbilical wall) may affect the amplitude of ornamentation: one can see that the out-of-plane aperture undulation in Fig. 2, is located more dorsally (closer to the umbilical wall rather than at mid-flank) on the right side (Fig. 2b) than on the left side (Fig. 2a) and is associated with a more conspicuous local kick in the curvature of the sinuous ribs and the presence of tubercles. Rudraraju et al. [79] showed how the local curvature of the shell margin may influence the three dimensional folding of the mantle and hence the location and amplitude of ornamental features such as spines ([79], figs. 10, 11). We expect the same to be true for commarginal ribs.

Hence, a potential explanation for the asymmetry of the Cénaret specimen would be an early asymmetry in the length (or perhaps stiffness) of the lappet on the two sides. In analogy with the formation of spines and in agreement with our current understanding of mechanical control of morphogenesis of shell ornamentation, we suggest that the more the growth lines extend out-of-plane in the growth direction, the deeper the lateral groove should be.

Regardless of the proximal cause of its asymmetry, the Cénaret specimen illustrates in our view the close developmental relationship between ammonoids that display strong sinuous commarginal ribs with a relatively smooth flank and those that display fainted sinuous ribs and a pronounced longitudinal groove. A continuous variation among these characters could be associated with the length of a longitudinal lappet-like extension of the mantle resulting in more or less out-of-plane apertures with varying curvatures. In this scheme, we do not expect that the evolutionary transition from sinuous ribs to lateral grooves is more frequent than the transition from lateral grooves to sinuous ribs. We suggest however that taxa with deep lateral grooves necessarily have an ancestor with out-of-plane apertures, possibly with sinuous ribs.

## Conclusions

We defined a new forma-type pathology, “forma janusa”, for specimens that display a marked left-right asymmetry of the shell matching two distinct, known species, in the absence of any clear evidence for injury or parasitism. We suggest that “forma janusa” specimens reflect an underlying developmental plasticity that potentially helped to fuel the rapid diversification of many ammonoid clades. We hypothesize that such “forma janusa” specimens may also reflect unsuspected phylogenetic closeness between the two displayed species and may even reflect a direct ancestor-descendant relationship. The study of the pathological specimen from Cénaret further leads us to suggest that the genera *Hildoceras* and *Haugia*/*Brodieia* are phylogenetically much closer than previously expected. We also point out that this specimen shows a left-right asymmetry in both ornamental characters and suture line characters corresponding accurately to two known species. This supports the view that ornamentation and suture line characters are not strictly independent, as generally considered in regular taxonomic practice.

In our view, pathologies also reflect the ‘normal’ developmental mechanisms of these extinct animals. We thus suggest that there is potentially a way to bridge the apparent morphological gap between sinuous ribs and longitudinal grooves in ‘normal’ development. These two characters would be dependent on the presence of an out-of-plane aperture as evidenced directly by growth lines and indirectly by the presence of sinuous ribs, showing mantle lappet-like extensions in the growth direction. By extrapolating the results of recent mechanical models of ornamental features, the deepness of the longitudinal grooves could be a consequence of continuous variation in the length of these lappets. This hypothesis is compatible with observations on ammonoids, gastropods and bivalves showing that the mantle edge is varying in length in the growth direction among variously ornamented specimens. This hypothesis is also compatible with recent morpho-mechanical models of the morphogenesis of ornamentation. In this scheme, we do not expect that the transition from sinuous ribs to lateral grooves is more frequent than the transition from lateral grooves to sinuous ribs. We suggest however that taxa with deep lateral grooves necessarily have an ancestor with an out-of-planar aperture with at least a short mantle lappet-like extension and possibly sinuous ribs.

As stated by Alberch [89], this study exemplifies that “monsters are a good system to study the internal properties of generative rules. They represent forms which lack adaptive function while preserving structural order. An analysis of monsters, is a study of "pure form" in the classical Naturphilosophie sense. There is an internal logic to the genesis and transformation and in that logic we may learn about the constraints on the normal.”

## Supplementary information

**Additional file 1. **Scanning Electron Microscope (SEM) picture of the inner whorls and the protoconch of the left side assigned to *Hildoceras semipolitum*.

**Additional file 2. **Scanning Electron Microscope (SEM) picture of the inner whorls and the protoconch of the right side assigned to *Brodieia primaria*.

## Data Availability

The Cénaret specimen and the specimen from Tintant [33] are held at the University of Burgundy, Dijon, France (specimen numbers: UBGD 28012 and UBGD 277447, respectively).
