# Supplementary figures and images for: Learning from beautiful monsters: phylogenetic and morphogenetic implications of left-right asymmetry in ammonoid shells
Source: BMC Evol Biol. 2019 Nov 13;19:210. doi: 10.1186/s12862-019-1538-5 (PMC6854895; doi:10.1186/s12862-019-1538-5)

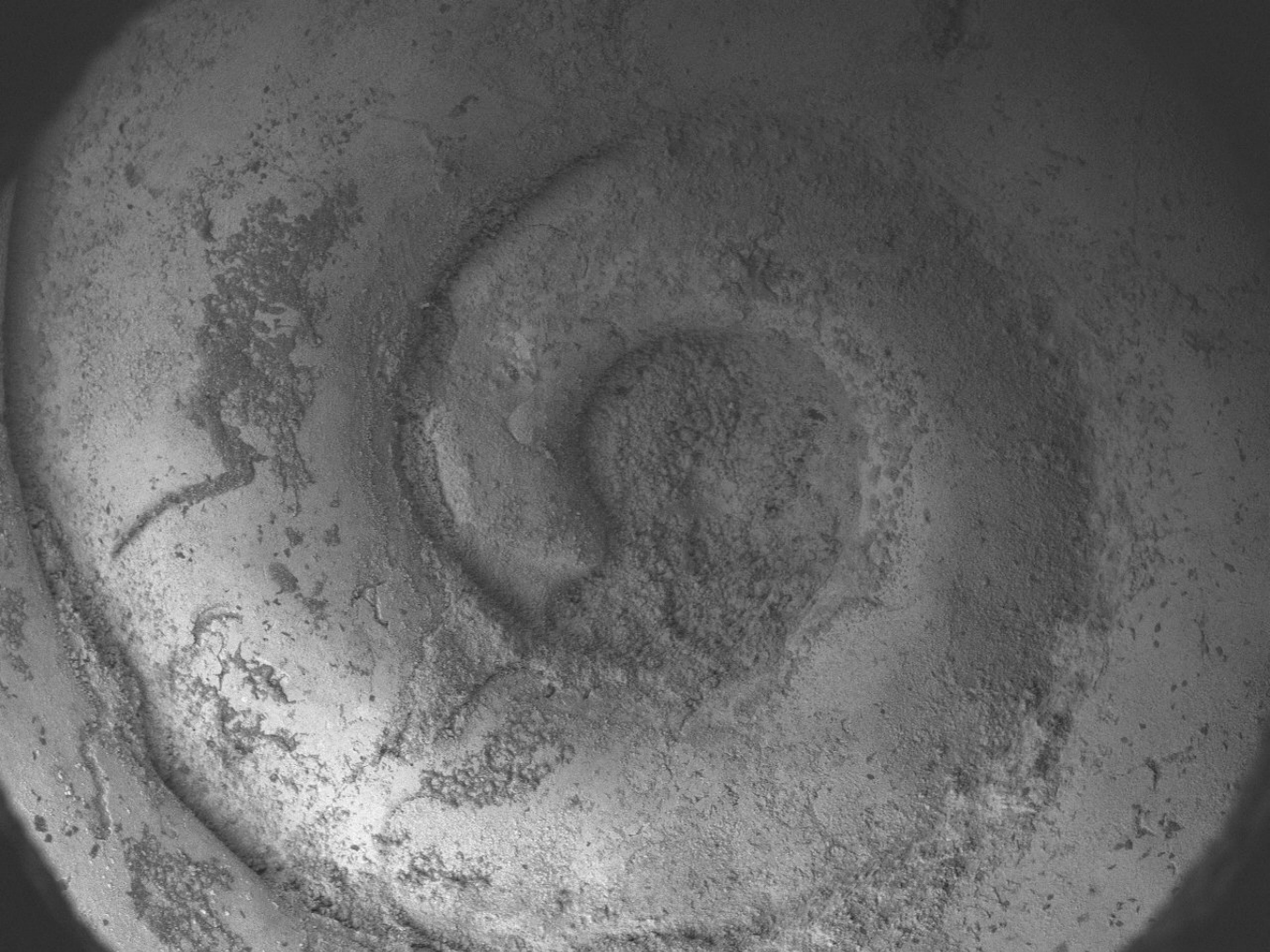

Gly0041

S D4,3 x40

2 mm

Supplement: Supplementary file 1 — Additional file 1. Scanning Electron Microscope (SEM) picture of the inner whorls and the protoconch of the left side assigned to Hildoceras semipolitum. [file 12862_2019_1538_MOESM1_ESM.pdf]

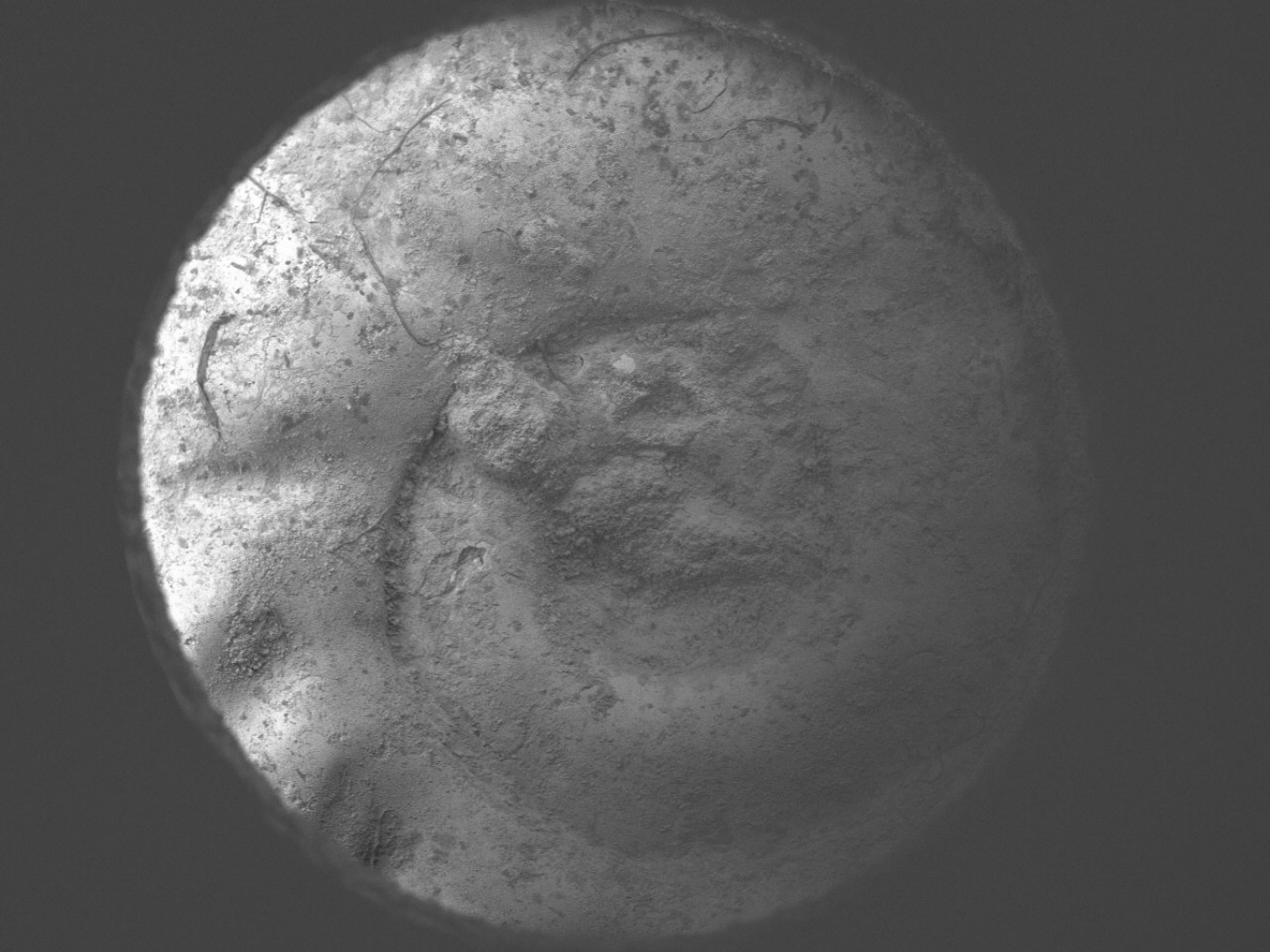

Gly0036

S D3,2 x30

2 mm

Supplement: Supplementary file 2 — Additional file 2. Scanning Electron Microscope (SEM) picture of the inner whorls and the protoconch of the right side assigned to Brodieia primaria. [file 12862_2019_1538_MOESM2_ESM.pdf]
